# Bilateral, Symmetrical, Congenital Becker’s Nevus Melanosis: A Rare Presentation and Review of the Literature

**DOI:** 10.7759/cureus.34594

**Published:** 2023-02-03

**Authors:** Rahaf A Abdulwahab, Tasneem A Banjar, Emad A Alharbi

**Affiliations:** 1 Medicine and Surgery, Umm Al-Qura University, Makkah, SAU; 2 Dermatology, King Abdulaziz Hospital, Makkah, SAU

**Keywords:** becker’s nevus, becker’s melanosis, becker’s nevus syndrome, rare case, congenital, case report, bilaterally

## Abstract

Becker’s nevus, also known as Becker’s melanosis or Becker’s pigmentary hamartoma, is a concurrent melanosis first described by S. William Becker. It is a type of acquired hyperpigmentation characterized by well-defined, unilateral lesions with regular borders. It is associated with hypertrichosis and hyperpigmented brownish patches with a mean diameter of 15 cm. The shoulder, scapular area, and upper arms are the most commonly affected areas, but it can occur on any area of the body, including the forehead, face, neck, lower trunk, extremities, and buttocks. The lesion usually appears around puberty, and males are more likely to be affected than females.

A 27-year-old male of Arabic descent who was medically free presented to the dermatology clinic complaining of bilateral, symmetrical, hyperpigmented patches on the upper back. The lesions started almost at birth, gradually growing in size and darkening in color over time. On local skin examination, the lesions were bilateral, symmetrical, hyperpigmented patches on the upper back. They were both homogeneous and brown in color with irregular borders and blotchy hyperpigmented macules on both sides of the upper back associated with sparse hair development. Histopathological examination revealed epidermal hyperkeratosis, acanthosis, and focal regular elongation of rete ridges with clubbing. Increased basal layer pigmentation was noticed. The dermis showed focal areas with pigment incontinence. Based on the above clinicopathological findings, the patient was diagnosed with Becker’s melanosis. He was referred to the laser clinic for further treatment.

## Introduction

Becker’s nevus (BN), also known as Becker’s melanosis or Becker’s pigmentary hamartoma, is a concurrent melanosis first described by S. William Becker. It is a type of acquired hyperpigmentation characterized by well-defined, unilateral lesions with irregular borders. It is associated with hypertrichosis and hyperpigmented brownish patches with a mean size of 15 cm. Although it occurs mostly on the shoulders, scapular area, and upper arms, it can also be found on any area of the body, including the forehead, face, neck, lower trunk, extremities, and buttocks. It is rare when it appears as a bilateral lesion or in unusual locations. Males are more prone to be affected than females, with a ratio of 5:1 and a prevalence of 0.5%. The lesion generally appears around puberty; however, there have been a few reports of BN appearing at birth or in early infancy. The majority of the cases are sporadic, whereas some are familial [1]. It is associated with hypertrichosis that generally occurs after hyperpigmentation has been established. Furthermore, BN occurs in combination with ipsilateral muscular and skeletal defects such as breast hypoplasia, scoliosis, vertebral defects, and hypoplasia of the muscles in the upper trunk known as BN syndrome (BNS) [1,2]. It is one of the epidermal nevus syndromes which is a broad phrase to describe epidermal nevi in association with syndromic features and is quite rare. These connected syndromes share cutaneous, neurologic, skeletal, and ophthalmologic features [2]. Only a few cases of multiple and bilateral BN have been described in the literature [3]. Therefore, we present the case of a 27-year-old healthy male with congenital, atypical, bilateral, symmetrical BN over the upper back.

## Case presentation

**Figure 1:**
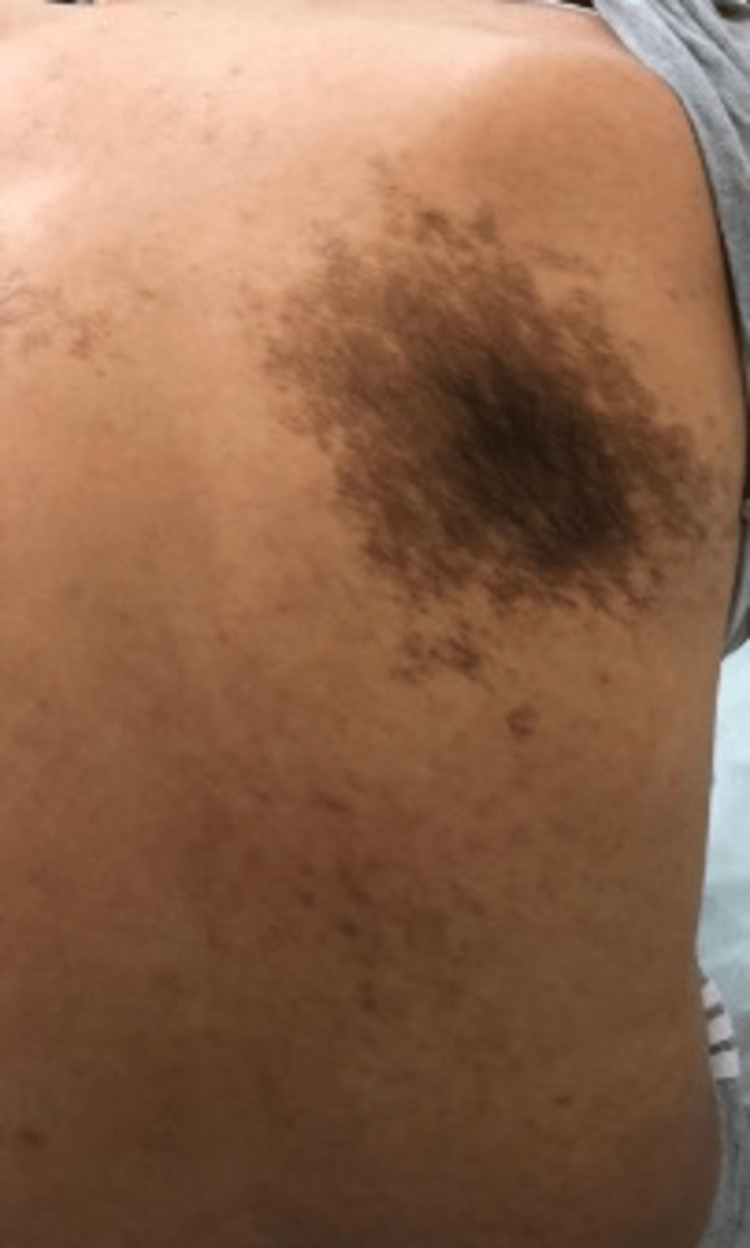
Becker’s nevus on the right side.

**Figure 2:**
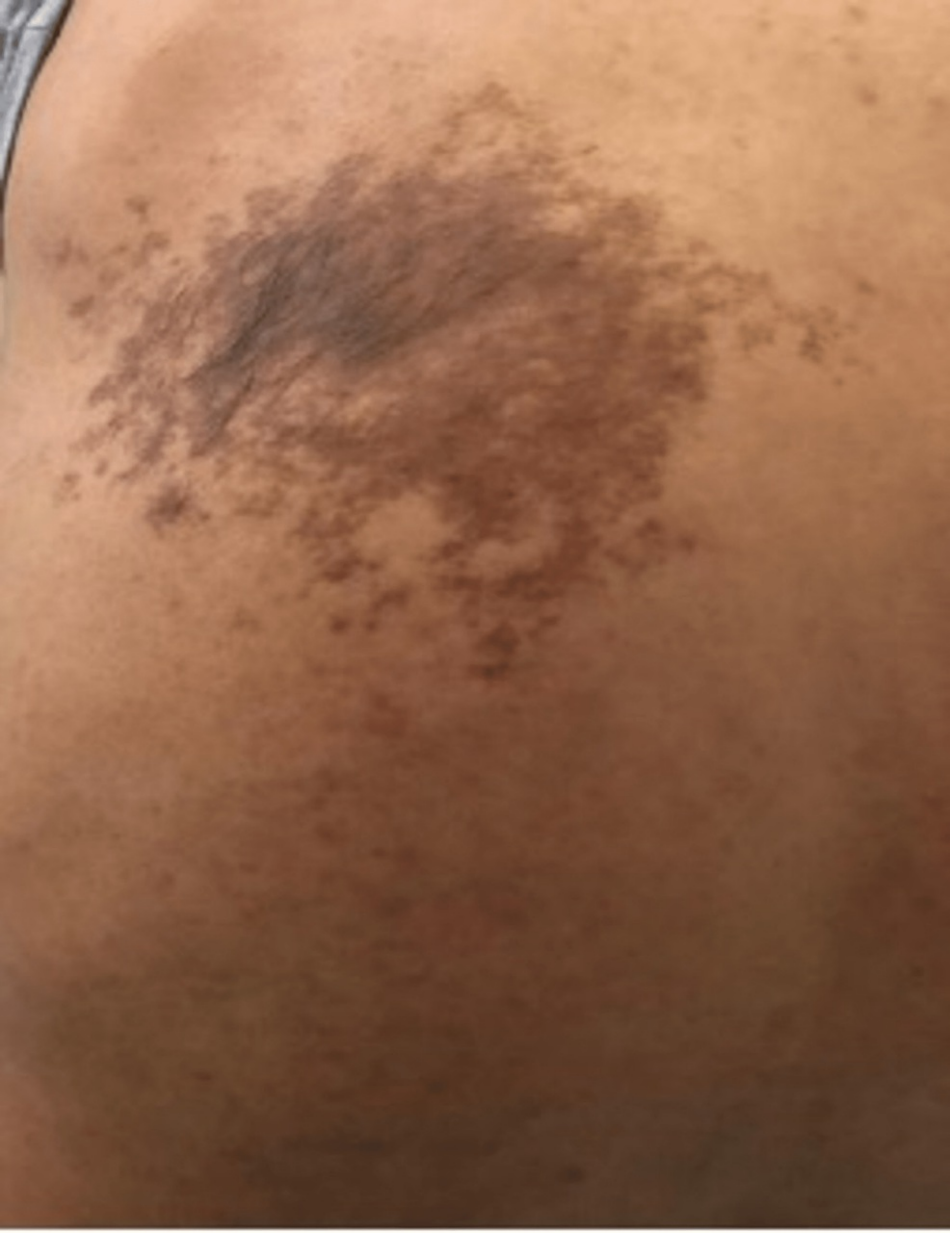
Becker’s nevus on the left side.

**Figure 3:**
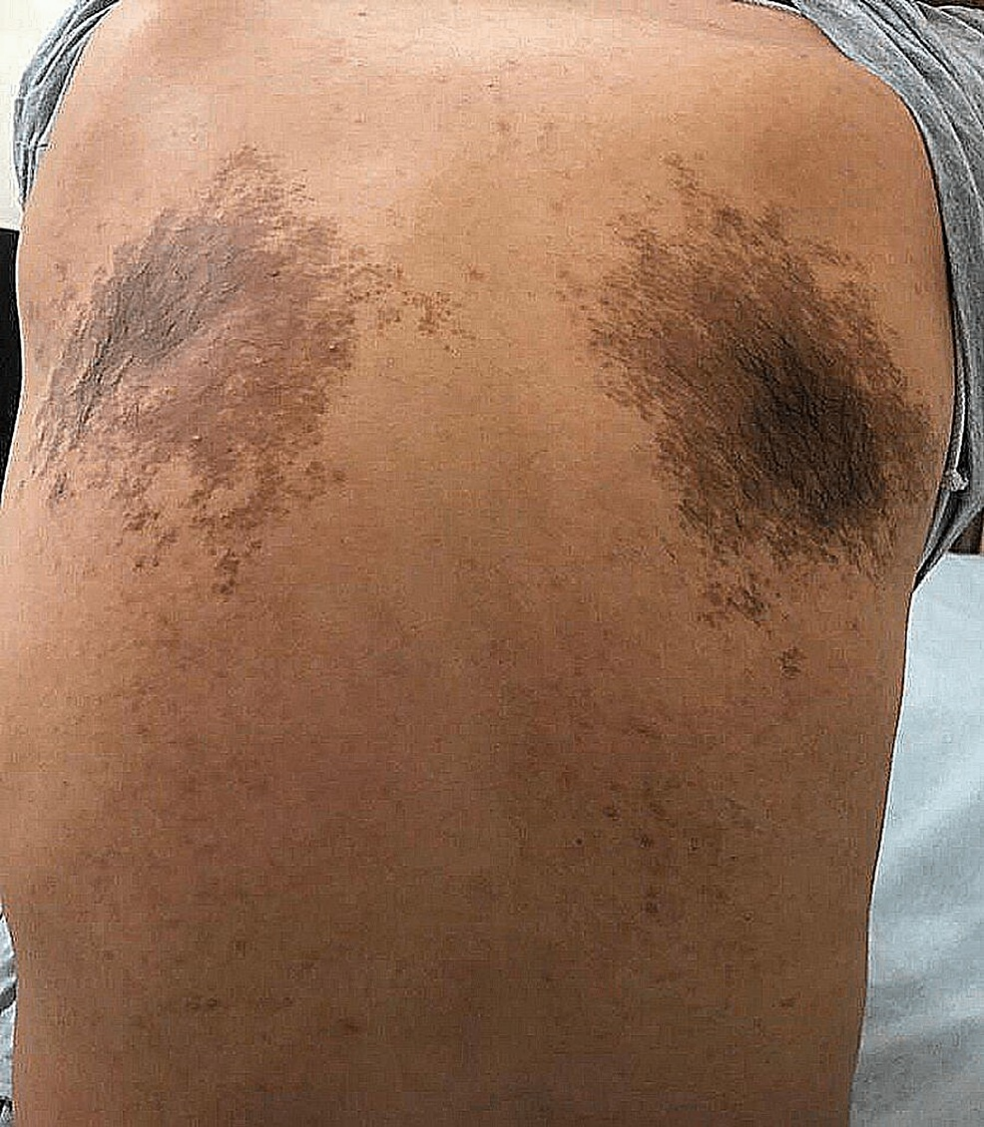
Well-defined bilateral hyperpigmentation with irregular borders on the upper back of the patient.

**Figure 4:**
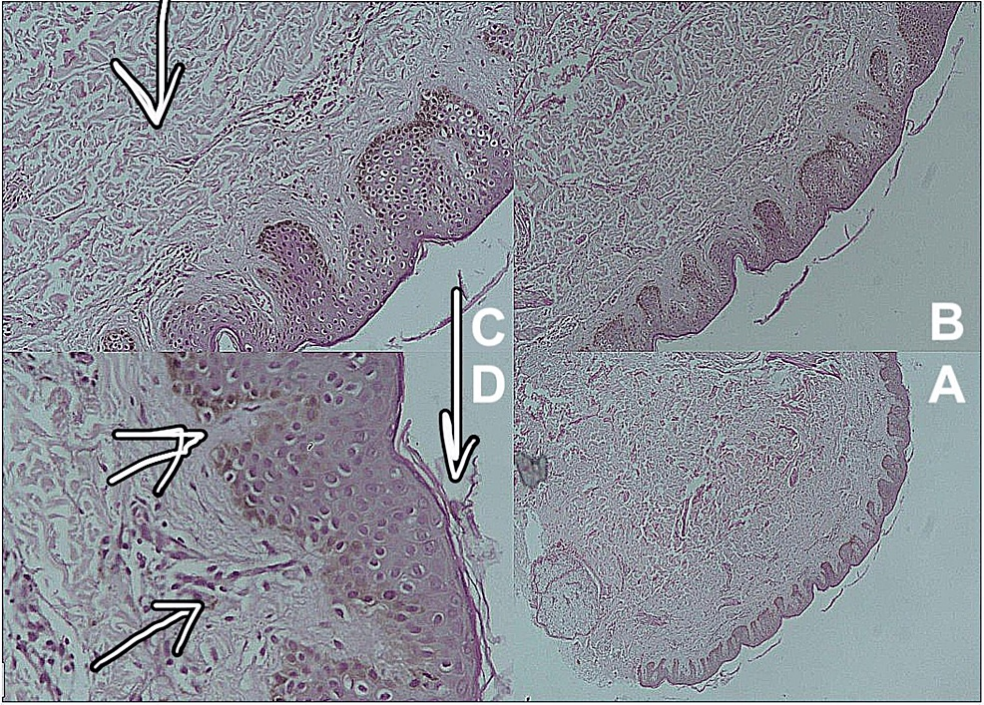
The histopathology pictures, epidermal hyperkeratosis, acanthosis, and focal regular elongation of rete ridges with clubbing. Increased basal layer pigmentation is noticed. The dermis shows focal areas with pigment incontinence.

A 27-year-old healthy male of Arabic descent presented to the dermatology clinic complaining of bilateral, symmetrical, hyperpigmented patches on the upper back. The lesions started almost at birth with a gradual increase in size and darkening in color over years and were associated with the development of hypertrichosis on the lesions. There were no relieving or aggravating factors and no associated pain, itching, bleeding, or ulceration. A review of the systems and past medical, surgical, drug, and allergy history were all unremarkable. There was no family history of any skin diseases or medical or congenital conditions. The patient’s four siblings were normal, with no active or previous complaints or skin manifestations. The patient’s antenatal history included normal vaginal delivery with no complications, as well as full-term and up-to-date immunization records. The mother was healthy, and she did not receive any medications during pregnancy. Body, hair, nails, and mucous membrane examinations were normal, and the patient had not previously received any treatment for this lesion and had not sought medical care. On local skin examination, the lesions were bilaterally and symmetrically hyperpigmented patches on the upper back, with the right one measuring 15 × 7 cm (Figure 1) and the left one measuring 17 × 9 cm in diameter (Figure 2). Both were homogeneous and brown in color with irregular borders and blotchy hyperpigmented macules on either side of the upper back, and they were associated with sparse hair development (Figure 3). There was no difference in skin texture compared to normal skin. A 4-mm skin punch biopsy was obtained from the back. Histopathological examination revealed epidermal hyperkeratosis, acanthosis, and focal regular elongation of rete ridges with clubbing. There was increased basal layer pigmentation. The dermis revealed focal areas with pigment incontinence. There was no increase in smooth muscle in the dermis or hyperplasia of the hair follicles (Figure 4). Chest X-ray, spine X-ray, and abdominal ultrasound were done to rule out systemic involvement, and they were all unremarkable. Based on the above clinicopathological findings, the patient was diagnosed with Becker’s melanosis. He was referred to the laser clinic for further treatment.

## Discussion

BN lesions may develop for a year or two after they first appear before becoming fixed in size. Pigmentation fades with time, but associated hypertrichosis persists. The specific genetic basis of BN is unknown. Given the mosaic/regional distribution of BN and the presence of sporadic and familial instances, a dominant mechanism of inheritance is proposed [1]. According to the literature, there is a disturbance in the androgen receptor activity in BN, as evidenced by increased androgen receptor density [4,5]. Our case represents a congenital, bilateral, symmetrical, atypical BN in a male of Arabic descent, not associated with syndromic features, which is a rare presentation. However, the histopathological features were typical for BN. Bilateral BN has not yet been documented in Middle Eastern countries; to our knowledge, only one case has been published by Alhuqayl et al. Table 1 summarizes a literature review of the published case reports with a similar presentation. To our knowledge, previous recent published studies reported a few cases of symmetrical or asymmetrical bilateral presentation [3-5]. Multiple and bilateral BN is rarely reported in the literature, and there are few reported cases in Arabic countries. BN in combination with ipsilateral muscular and skeletal defects such as breast hypoplasia, scoliosis, vertebral defects, and hypoplasia of the muscles in the upper trunk is known as BNS [1,2]. BNS is most commonly found around the anterior upper trunk, with or without hypertrichosis and/or acneiform lesions. Our patient had bilateral, symmetrical BN of both scapular regions. However, a neurological, genital, and limb examination revealed no abnormalities, and abdominal ultrasound and skeletal radiographic imaging revealed no abnormalities. Our differential diagnosis included *cafe au lait* macules, congenital melanocytic nevus, congenital smooth muscle hamartoma, and plexiform neurofibroma. All were excluded because of the presence of clinical and histopathological features of BN. The therapeutic modalities were limited and mainly indicated for cosmetic reasons. Various types of lasers have been shown to be effective in BN. Two types of lasers are mostly used, namely, Q-switched ruby lasers and Q-switched Nd-YAG lasers. However, both are associated with a high recurrence rate [1]. Our patient was referred to the laser clinic for further treatment.

**Table 1:** Summary of a literature review of seven cases of Becker’s nevus.

Author	Year	Sex	Age (years)	Duration	Site	Congenital/Acquired
Current report	2022	Male	27	Since birth	Both scapular regions	Congenital
Rao [4]	2021	Male	23	Since birth	Both scapular and suprascapular regions, both the shoulders, arms, and the extensor aspect of forearms	Congenital
Alhuqayl et al. [3]	2019	Female	20	Four years	Over the upper back, chest, and breasts	Acquired
Yeşilova et al. [6]	2013	Male	16	Five years	Abdominal region	Acquired
Grim et al. [7]	2009	Male	45	Adolescence	The front of the chest	Acquired
Khatami et al. [8]	2008	Male	14	Six years	Both scapular regions, anterior chest, and upper arm	Acquired
Ferreira et al. [9]	1998	Female	4	Since birth	Scapular region, shoulders, and arms	Congenital

## Conclusions

BN is a type of acquired hyperpigmentation characterized by a well-defined, unilateral lesion with irregular borders. It is associated with hypertrichosis and hyperpigmented brownish patches. It can be found in any area of the body, but it is rare when it appears as a bilateral lesion. Our case represents a congenital, bilateral, symmetrical, atypical BN not associated with syndromic features, which is a rare presentation. The purpose of reporting this case is to increase the understanding of the various manifestations of this disease.
